# Vesnarinone downregulates CXCR4 expression via upregulation of Krüppel-like factor 2 in oral cancer cells

**DOI:** 10.1186/1476-4598-8-62

**Published:** 2009-08-12

**Authors:** Daisuke Uchida, Tomitaro Onoue, Nasima-Mila Begum, Nobuyuki Kuribayashi, Yoshifumi Tomizuka, Tetsuya Tamatani, Hirokazu Nagai, Youji Miyamoto

**Affiliations:** 1 Department of Oral Surgery, Subdivision of Molecular Oral Medicine, Division of Integrated Sciences of Translational Research, Institute of Health Biosciences, The University of Tokushima Graduate School, 3-18-15 Kuramoto, Tokushima, Japan

## Abstract

**Background:**

We have demonstrated that the stromal cell-derived factor-1 (SDF-1; CXCL12)/CXCR4 system is involved in the establishment of lymph node metastasis in oral squamous cell carcinoma (SCC). Chemotherapy is a powerful tool for the treatment of oral cancer, including oral SCC; however, the effects of chemotherapeutic agents on the expression of CXCR4 are unknown. In this study, we examined the expression of CXCR4 associated with the chemotherapeutic agents in oral cancer cells.

**Results:**

The expression of CXCR4 was examined using 3 different chemotherapeutic agents; 5-fluorouracil, cisplatin, and vesnarinone (3,4-dihydro-6-[4-(3,4-dimethoxybenzoyl)-1-piperazinyl]-2-(1H)-quinolinone) in B88, a line of oral cancer cells that exhibits high levels of CXCR4 and lymph node metastatic potential. Of the 3 chemotherapeutic agents that we examined, only vesnarinone downregulated the expression of CXCR4 at the mRNA as well as the protein level. Vesnarinone significantly inhibited lymph node metastasis in tumor-bearing nude mice. Moreover, vesnarinone markedly inhibited 2.7-kb human CXCR4 promoter activity, and we identified the transcription factor, Krüppel-like factor 2 (KLF2), as a novel vesnarinone-responsive molecule, which was bound to the CXCR4 promoter at positions -300 to -167 relative to the transcription start site. The forced-expression of KLF2 led to the downregulation of CXCR4 mRNA and impaired CXCR4 promoter activity. The use of siRNA against KLF2 led to an upregulation of CXCR4 mRNA.

**Conclusion:**

These Results indicate that vesnarinone downregulates CXCR4 via the upregulation of KLF2 in oral cancer.

## Background

Worldwide, oral cancer, excluding pharyngeal cancer, represents 2.65% of all human malignancies [1]. Most oral cancer is histopathologically diagnosed as SCC. Oral cancers, including OSCC are characterized by a high degree of local invasion and a high rate of metastases to the cervical lymph nodes. In particular, it has been demonstrated that lymph node metastasis directly affects the prognosis of patients with OSCC as a results of extracapsular spread and induces the secondary distant metastasis [2,3]. Lymph node metastasis is frequently detected upon the first visit to a hospital, but it also appears during initial treatments that include radiotherapy and chemotherapy. Lymph node metastasis has been primarily treated by surgical resection, although pre- or post-operative chemotherapy and/or pre- or post-operative radiotherapy have been reported to be useful for achieving a favorable course [4,5]. Despite the evidence accumulated to date, there is no known molecular target for anti-metastatic therapy against nodal involvement patients with oral cancer.

Chemokines are a large family of small (7–15 kDa), structurally related, heparin-binding proteins that have been identified as attractants of different types of blood leukocytes to sites of infection and inflammation [6]. They are produced locally in various tissues and act on leukocytes via selective membrane-bound G protein-coupled receptors, the two major subfamilies of which are CCR and CXCR. Among these chemokines and their receptors, the SDF-1 (also referred to as CXCL12)/CXCR4 system has been shown to be involved in both lymph node and distant metastases of several types of cancer [7-12]. We have also demonstrated that lymph node metastatic OSCC cells specifically express functional CXCR4, and the SDF-1/CXCR4 system is involved in lymph node metastatic processes in OSCC [13,14]. Moreover, we recently revealed that the expression of CXCR4 in OSCC at the primary site was significantly associated with lymph node metastasis, mode of invasion, tumor recurrence, and patient prognosis [15]. Recently, we have demonstrated that, in cases of oral SCC, the paracrine SDF-1/CXCR4 system potentiates lymph node metastasis, but distant metastasis might require the autocrine SDF-1/CXCR4 system [16].

Considering the importance of CXCR4 in a variety of cancers, attempts have been made to blockade this molecule as a therapeutic target. For example, the administration of CXCR4-neutralizing antibody into tumor-bearing mice by Müller and colleagues inhibited lymph node and lung metastasis in a breast cancer metastasis model [7]. Furthermore, CXCR4 has been shown to function as a co-receptor for T cell line-tropic HIV-1 entry [17]; and for this reason, there has been considerable interest in the clinical potential of CXCR4 inhibitors such as bicyclam AMD3100 [18], horseshoe crab-derived peptide T22 [19], and the non-peptide TAK-779 [20] for inhibiting HIV infection. However, the clinical application of such inhibitors must be approached with caution, in light of the importance of the CXCR4 pathway in normal lymphohematopoiesis [21]. Moreover, for the effective treatment of cancer, compounds with growth inhibitory effects as well as anti-metastatic effects are desirable. Therefore, if conventional chemotherapeutic agents that exert inhibitory effects on CXCR4 are identified, it is expected that anti-metastatic therapy could be performed more safely and effectively. However, the molecular mechanism(s) of diverse chemotherapeutic agents against CXCR4 expression are not yet fully understood. Thus, in this study, we examined the transcriptional regulation of CXCR4 by chemotherapeutic agents in an oral cancer cell line, B88, which expresses high levels of CXCR4, and which exhibits lymph node metastatic potential *in vivo*.

## Methods

### Reagents and materials

For *in vitro *experiments, vesnarinone (OPC-8212; Otsuka Pharmaceutical Company, Tokyo, Japan) was dissolved in DMSO at concentration of 10 mg/ml as the first stock solution, and the first stock solution was diluted with the complete culture medium [22]. For *in vivo *experiment, vesnarinone was suspended in 0.5% CMC (Daiichi seiyakukogyo, Kyoto, Japan) [23]. 5-FU (Sigma, St. Louis, MO) and CDDP (Sigma) was dissolved in DMSO at concentration of 1 mg/ml, and diluted with the complete culture medium at 10 μg/ml. Several CXCR4 promoter luciferase construct was kindly provided by Drs. Peter Staller and Wilhelm Krek (Friedrich Miescher Institute for Biomedical Research, Basel, Switzerland). KLF2 expression vectors (pcDNA3-KLF2, pcDNA3-Δ KLF2, and pGEX-4T-1-KLF2) were generous gifts from Drs. Sucharita Banerjee and Mukesh K Jain (Cardiovascular Division, Brigham and Women's Hospital, Boston, MA).

### Mice

BALB/c nude mice were purchased from CLEA Japan (Osaka, Japan). The mice were maintained under pathogen-free conditions and were handled in accordance with the Guidelines for Animal Experimentation of Tokushima University. The experiments were initiated when the mice were 8 weeks of age and were performed as described previously [24]. The experimental chemotherapy by use of vesnarinone was performed as described previously with slight modification [23]. Briefly, B88 cells constitutively expressing firefly luciferase (B88-luc) were orthotopically inoculated into masseter muscle of nude mice. One week later, the mice were treated by the daily oral administration of 0.5% CMC (5 ml/kg) for a vehicle or 0.2 ml of 10 mg/ml vesnarinone (50 mg/kg). The tumor volume was estimated by measuring tumor size and using the following formula: tumor volume = 1/2 × L × W^2^, where L and W represent the largest diameter, and the smallest diameter, respectively. The presence or absence of lymph node and distant metastases was confirmed by the H&E staining.

### Cells and cell culture

High pulmonary metastases clone ACC-M was selected from cell line ACC-2 that was derived from surgically excised primary tumor tissue from a histologically diagnosed patient with adenoid cystic carcinoma of the palate [25]. ACC-M and B88 cells [13] were maintained in RPMI-1640 and DMEM, respectively, supplemented with 10% fetal calf serum (FCS), 100 μg/ml streptomycin, and 100 U/ml penicillin in a humidified atmosphere of 95% air and 5% CO_2 _at 37°C. ACC-M cell lines were supplied friendly from Drs. Weiliu Qiu and Wantao Che (Laboratory of Tumor Biology, Ninth People's Hospital, Shanghai Jiao Tong University, School of Stamotology).

### Quantitative RT-PCR

After stimulation of the cells with or without chemotherapeutic agents, the preparation of total RNA and reverse transcription were performed as described previously [15]. In quantitative PCR, CXCR4 and KLF2, CXCR7 or GAPDH mRNA were detected with QuantiTect™ Gene Expression Assays (Hs-CXCR4; Qiagen, Hilden, Germany) and Taqman™ Gene Expression Assay (Applied Biosystems, Foster City, CA), respectively. Gene-specific products were measured continuously by an ABI PRISM 7000 Sequence Detection System (Applied Biosystems) during 40 cycles. Experiments were performed at least three times.

### Flow cytometric analysis

Logarithmically growing oral cancer cells were trypsinized and fixed in 4% (w/v) paraformaldehyde on ice for 10 min. The cells were washed and incubated with anti-human CXCR4 mAb (dilution 1:100, 12G5; BioSource International, Inc. Camarillo, CA) or mouse IgG isotype control (SouthernBiotech, Birmingham, CA) for 30 min at room temperature. After being washed twice with PBS, the cells were incubated with PE-labeled goat anti-mouse IgG (Serotec, Sapporo, Japan) for 30 min at room temperature and analyzed with an EPICS flow cytometer (Coulter, San Jose, CA).

### *In vitro *cell migration assay

The *in vitro *migration of B88 cells was evaluated using the Transwell (CORNING, Corning, NY) as described previously [13]. In some experiments, cells were incubated with vesnarinone (50 μg/ml) or were transfected with KLF2 expression vectors before the addition of cells in the upper chamber.

### Construction of CXCR4 promoter-luciferase reporter plasmids

**Figure 1:**
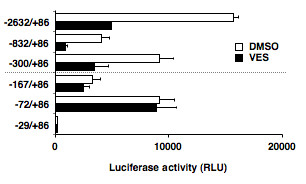
**Inhibition of luciferase activity in the presence of vesnarinone**. Several CXCR4-luciferase constructs were transfected into B88 cells, which were treated with or without vesnarinone for 48 h, and then luciferase activities were measured.

Several truncated promoter constructs (Figure 1) were generated by restriction digestion of pGL2-CXCR4 (-832/+86) with *Kpn*I and *Mlu*I for pGL2-CXCR4 (-300/+86), with *Kpn*I and *Sac*II for pGL2-CXCR4 (-29/+86), or with *Kpn*I and *Xho*I for pGL2-CXCR4 (0). The digested fragments were treated with T4 DNA polymerase (Takara Biomedicals, Kusatsu, Japan) and self-ligated with T4 DNA ligase (Takara Biomedicals). pGL2-CXCR4 (-167/+86) and pGL2-CXCR4 (-72/+86) were generated by PCR, using CXCR4-UP (5'-GGGGTACCCAAACTTAGGAAATGCCTCTGGGA-3') and CXCR4-DN1 (5'-GGGGTACCAGCGGCGCATGCGCCGCGCTCGGAGCG-3'), and CXCR4-UP and CXCR4-DN2 (5'-CCGCTCGAGCCTCCATGGTAACCGCTGGT-3'), respectively.

### Transcriptional regulation of CXCR4 promoter by vesnarinone

B88 or ACC-M cells (5 × 10^5 ^cells/dish) were co-transfected with 5 μg of CXCR4-luciferase reporter vectors and 0.5 μg of pcDNA3 (Invitrogen, Carlsbad, CA) by electroporation (140 V, 99 msec, 1 pulse), using ELECTRO SQUARE PORATOR T820 (BTX Inc., San Diego, CA). After transfection, stable transfectants were isolated and the cells were treated with or without 50 μg/ml of vesnarinone as indicated. Then, the cells were lysed with cell culture lysis reagent (Pikkagene, Toyo Ink, Tokyo, Japan), and luciferase activity was measured by a Lumat LB9507 (BERTHOLD TECHNOLOGIES, Wildbad, Germany) using a luciferase assay kit (Toyo Ink) according to the manufacture's instruction. The luciferase activity was normalized by protein quantitation. Each experiment was repeated at least twice.

### cDNA microarray

Total RNA for cDNA microarray analysis was extracted from B88 cells treated with or without vesnarinone for 48 h. cDNA microarray was performed and analyzed in Hokkaido System Science (Sapporo, Japan) using a human 1A Oligo Array (Agilent Technologies, Palo Alto, CA).

### Chromatin immunoprecipitation (ChIP) assay

B88 cells were treated with or without vesnarinone for 48 h, or were transfected with pcDNA3 or pcDNA3-KLF2. ChIP assay was performed by using a ChIP assay kit according to the manufacture's instruction (upstate, Lake Placid, NY). Briefly, equal aliquots of isolated chromatin DNA were subjected to immunoprecipitation with a rabbit KLF2 antibody (Santa Cruz Biotechnology, Santa Cruz, CA). The DNA fragments associated with specific immunoprecipitates were purified and used as templates for the PCR in order to amplify the CXCR4 promoter sequences containing a CACCC/KLF binding site (-401 to -142). The primers used were 5'-CAGCAAGTCACTCCC-3' and 5'-GGAGAGGTGCGCGGC-3'.

### Electrophoretic mobility-shift assay (EMSA)

Biotinylated double stranded oligonucleotides of CXCR4 promoter sequence at positions -204 to -175 containing the CACCC site (5'-ACTCACTACCGAC**CACCC**GCAAACAGCAGG-3') or a mutated CACCC site (5'-ACTCACTACCGAC**TGTTT**GCAAACAGCAGG-3') were synthesized in Hokkaido System Science. After transformation of glutathione S-transferase (GST) or GST-KLF2 plasmid vectors into BL21 (DE3) competent cells (Merck, Darmstadt, Germany), recombinant proteins were extracted, and were purified with glutathione sepharose column (Amersham Pharmacia Biotech.). EMSA was performed using LightShift Chemiluminescent EMSA Kit (Pierce, Rockford, IL) according to the manufacture's instruction.

### siRNA assay

siRNA against KLF2 was synthesized in B-Bridge international (Mountain View, CA). siRNA cocktails against KLF2 or negative control siRNA (B-Bridge International) were transfected into B88 cells, using Lipofectamine 2000 reagent (Invitrogen) at the final concentration of 20 nM or 200 nM. After 48-h incubation, total RNA was extracted and quantitative RT-PCR was performed.

### Statistical analysis

Statistical differences between the means for the different groups were evaluated with StatView 4.5 (Abacus Concepts, Berkeley, CA) using one-way ANOVA, with the level of significance at *P *< 0.05. All experiments were repeated two to three times, and similar Results were obtained.

## Results

### Expression of CXCR4 induced by the chemotherapeutic agents

**Figure 2:**
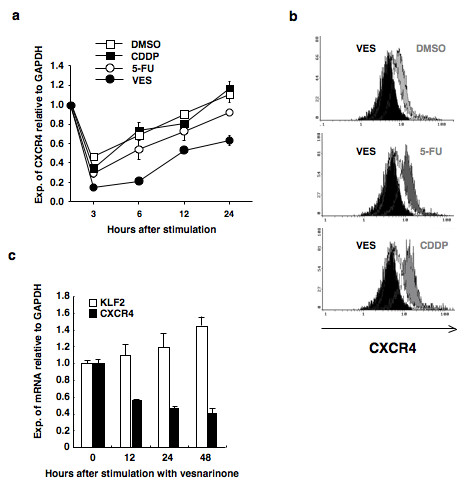
**Downregulation of CXCR4 by vesnarinone**. (a) CXCR4 mRNA was examined by quantitative PCR in B88 cells treated with chemotherapeutic agents (CDDP, 5-FU, or vesnarinone) or vehicle DMSO. (b) B88 cells treated with or without chemotherapeutic agents were incubated with or without anti-CXCR4 monoclonal antibody. Then the cells were incubated with PE-labeled goat anti-mouse IgG and analyzed by flow cytometry in order to determine the expression of CXCR4 protein. The white zones show the cells treated with vesnarinone stained by mouse IgG isotype control. The black zones show cells treated with vesnarinone, and gray zones show the cells treated with DMSO (*upper*), 5-FU (*middle*), or CDDP (*lower*), respectively. The black zones and gray zones were stained by anti-CXCR4 monoclonal antibody. (c) Vesnarinone downregulates CXCR4 mRNA and upregulates KLF2 mRNA in a time-dependent manner in ACC-M cells. ACC-M cells were treated with vesnarinone and quantitative RT-PCR was performed.

In complete medium or DMSO, the expression of CXCR4 mRNA was transiently downregulated after the medium was changed, but mRNA expression was gradually upregulated and recovered to the basal level at 24 h (Figure 2a). Initially, two conventional cytocidal chemotherapeutic agents for treating oral cancer, namely 5-FU and CDDP, were assessed, but the expression of CXCR4 mRNA was not altered with these treatments. Therefore, we tested the effect of a novel cytostatic chemotherapeutic agent, vesnarinone [22]. It was revealed that vesnarinone markedly downregulated the expression of CXCR4 mRNA and protein, as compared to the effects of DMSO, 5-FU, or CDDP in B88 cells (Figure 2a and 2b). Downregulation of CXCR4 mRNA by treatment with vesnarinone was also detected in oral cancer cells, ACC-M, in a time dependent manner (Figure 2c). Moreover, we also found the reduced expression of CXCR4 protein by the treatment with vesnarinone, as compared to the effects of 5-FU and CDDP both in ACC-M cells and in Hela cells (Additional File 1). Next, we examined the effect of vesnarinone on the expression of CXCR7, which was recently identified as a chemokine receptor that binds chemokines CXCL11 and SDF-1 [26]. However, analyses of cDNA microarray and quantitative RT-PCR revealed that vesnarinone was not found to change the expression of CXCR7 (data not shown).

### Effects of vesnarinone on the migration and lymph node metastasis of B88 cells

**Figure 3:**
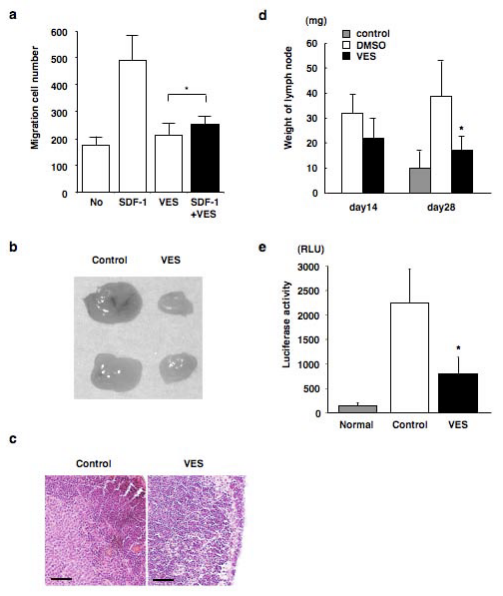
**Inhibition of migration and lymph node metastasis by vesnarinone in B88 cells**. (a) B88 cells were seeded on the upper membrane in the presence or absence of SDF-1α in the lower compartment, and in the presence of vesnarinone in the upper compartment. After 24 h, cells attached to the lower surface of the membrane were subjected to H&E staining and were counted. The bars show the SD of triplicate samples. Data are representative of three separate experiments with similar results. *, Not significant as determined by one-way ANOVA. (b, c, d and e) B88-luc cells (2 × 10^6^) were inoculated into the masseter muscle of nude mice, which were treated with or without vesnarinone and were sacrificed at day 14 (d) or day 28 (b, c, d and e). Extirpated lymph nodes (b), H&E staining of the lymph nodes (c) (bar, 50 μm), and the weight of lymph nodes (d) are shown. *, Significant as determined by one-way ANOVA (vs. controls, *P *< 0.01). (e) Lymph nodes were lysed and then luciferase activity was measured. *, Significant as determined by one-way ANOVA (vs. controls, *P *< 0.01).

In order to investigate the functional role played by CXCR4 downregulation with vesnarinone, an *in vitro *migration assay was performed. SDF-1 was found to enhance the migration of B88 cells, which was in contrast significantly inhibited by treatment with vesnarinone (Figure 3a). In order to further clarify the role of vesnarinone on lymph node metastasis, experimental chemotherapy was carried out. Vesnarinone significantly inhibited the size and weight of the metastatic lymph nodes in the orthotopic inoculation of B88 cells to nude mice (Figure 3b, c and 3d). Furthermore, when treated with vesnarinone, B88 cells constitutively expressing firefly luciferase showed significantly impaired luciferase activity in the metastatic lymph nodes (Figure 3e).

### Transcriptional regulation of the CXCR4 gene by vesnarinone

Since vesnarinone appeared to inhibit the lymph node metastasis by downregulating CXCR4, we investigated in greater detail the transcriptional regulation of CXCR4 by vesnarinone. A luciferase reporter vector driven by the 2.7-kb CXCR4 promoter region was transfected into B88 cells, and the stable transfectants were isolated. As shown in Additional File 2a, luciferase activities of the CXCR4 promoter, when treated with vesnarinone, were significantly lower than those of the promoter treated with DMSO vehicle alone. In order to further clarify the transcriptional regulatory region activated by vesnarinone in the CXCR4 promoter, we isolated several B88-transfectants with truncated CXCR4 promoter-luciferase constructs, and in those treated with vesnarinone, we observed an inhibition of luciferase activity driven downstream of the truncated CXCR4 promoters up to position -300 from the transcription starting site (Additional File 2b and 2c). However, the luciferase activity driven upstream of position -167 was not significantly impaired by treatment with vesnarinone (Additional File 2d, e, f and Figure 1). These results indicate that the vesnarinone-regulatory element on the CXCR4 promoter was located at positions -300 to -167 from the transcription starting site (Figure 1).

### Identification of vesnarinone-regulatory element on the CXCR4 promoter region

**Figure 4:**
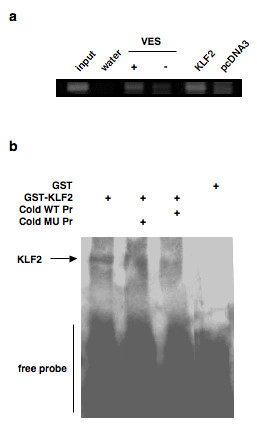
**KLF2 binding to the CXCR4 promoter via a CACCC site**. (a) ChIP assay. B88 cells were treated with or without vesnarinone for 48 h, or were transfected with pcDNA3 or pcDNA3-KLF2. Equal aliquots of isolated chromatin DNA were subjected to immunoprecipitation with rabbit KLF2 antibody, and the DNA fragments associated with specific immunoprecipitates were purified and used as templates for the PCR in order to amplify the CXCR4 promoter sequences containing the CACCC/KLF2 binding site (-401 to -142). (b) EMSA. Ten micrograms of recombinant GST or GST-KLF2 protein were incubated in the biotinylated CXCR4 promoter containing the CACCC site with or without an excess volume (200 fold) of non-labeled wild type probe (Cold WT Pr) or that of non-labeled mutated probe (Cold MU Pr).

Positions -300 to -167 contained several canonical transcription factor binding sites such as E-box, NF-κB, nuclear factor of activated T cells, and Krüppel-like factor (KLF). Therefore, we performed a microarray analysis to identify the vesnarinone-regulatory element on the CXCR4 promoter. Numerous molecules were upregulated or downregulated by treatment with vesnarinone, but the gene related to the vesnarinone-regulatory element and that with the highest upregulation detected was KLF2. To verify whether or not KLF2 binds to vesnarinone-regulatory element, we next performed a ChIP assay. After treatment with vesnarinone, KLF2 preferentially bound to vesnarinone-regulatory element (Figure 4a). Moreover, forced expression of KLF2 also led to binding to vesnarinone-regulatory element in the absence of vesnarinone stimulation, which indicated that KLF2 might regulate the expression of CXCR4 (Figure 4a). Next, we carried out an EMSA in order to confirm the binding of KLF2 to vesnarinone-regulatory element. Recombinant KLF2 effectively bound to the CXCR4 promoter sequence at positions -204 to -175 containing the CACCC site; however, the binding was not inhibited by the addition of excess volume of mutant probe (Figure 4b).

### Effects of KLF2 on the downregulation of CXCR4

**Figure 5:**
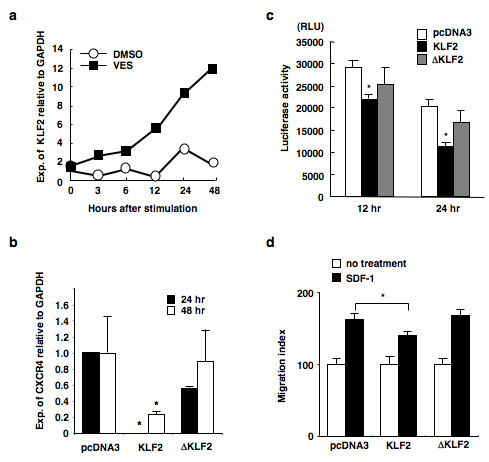
**Induction of KLF2 mRNA by vesnarinone and downregulation of CXCR4 by KLF2**. (a) Vesnarinone upregulates KLF2 mRNA in a time-dependent manner. B88 cells were treated with vesnarinone and quantitative RT-PCR was performed. (b) Forced expression of KLF2 downregulates CXCR4 mRNA. pcDNA3, KLF2 or ΔKLF2 expression vectors were transfected into B88 cells. After 48 h, quantitative RT-PCR was performed. (c) Forced expression of KLF2 impaired CXCR4 promoter activity. Stable transfectants of CXCR4 promoter-luciferase construct (-300/+86) were transfected with pcDNA3, KLF2, or ΔKLF2 expression vectors, and then luciferase activity was measured at the indicated time points. *, Significant as determined by one-way ANOVA (vs. pcDNA3, *P *< 0.01). (d) Forced expression of KLF2 impaired cell migration induced by SDF-1. B88 cells that had been transiently transfected with KLF2 expression vectors were seeded on the upper membrane in the presence or absence of SDF-1α in the lower compartment. After 24 h, cells attached to the lower surface of the membrane were stained by H&E and were counted. *, *P *< 0.05 (one-way ANOVA). The bars show the SD of triplicate samples. Data are representative of three separate experiments with similar results.

Next, we confirmed the upregulation of KLF2 by treatment with vesnarinone. Vesnarinone was found to upregulate KLF2 mRNA in a time-dependent manner both in B88 cells (Figure 5a) and in ACC-M (Figure 2c) cells. Therefore, we investigated the role played by KLF2 in the downregulation of CXCR4. Forced expression of KLF2 downregulated CXCR4 mRNA, but forced expression of ΔKLF2, with a deleted DNA binding domain, did not lead to such downregulation (Figure 5b). Moreover, forced expression of KLF2 also attenuated the luciferase activity driven by the CXCR4 promoter (-300/+76) both in B88 cells (Figure 5c) and in ACC-M cells (Additional File 3). Next, we examined the functional effects of KLF2 on the cell migration induced by SDF-1. In B88 cells, the migration induced by SDF-1 was significantly inhibited by the forced expression of KLF2, but this was not observed in the case of either pcDNA3 or ΔKLF2 (Figure 5d).

### Effects of silencing KLF2 on the expression of CXCR4

**Figure 6:**
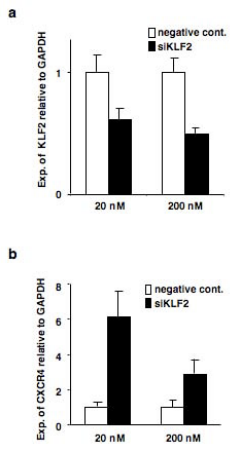
**Enhanced CXCR4 expression by siRNA against KLF2**. siRNA cocktails against KLF2 were transfected into B88 cells. After 48 h, quantitative RT-PCR against KLF2 (a) and CXCR4 (b) was performed. Data are representative of two separate experiments with similar results.

Since the overexpression of KLF2 induced the downregulation of CXCR4, we next attempted to examine the expression of CXCR4 using KLF2 knock-down and siRNA approaches. The introduction of the siRNA against KLF2 exhibited a 50% decrease in the KLF2 mRNA levels (Figure 6a), which in turn markedly upregulated CXCR4 mRNA (Figure 6b).

## Discussion

Vesnarinone, a synthetic quinolinone derivative with inotropic effects, was originally developed to treat cardiac failure [27] and has been clinically used for the treatment of patients with chronic congestive heart failure [28]. Vesnarinone was not developed as a targeted agent; however, recent studies have revealed that vesnarinone is a unique anti-proliferation [22,23], differentiation-inducing [29] and apoptosis inducing [30,31] agent against several human malignancies, including leukemia and several types of solid tumors. Furthermore, clinical studies have shown that vesnarinone potentiates the effects of conventional cytotoxic chemotherapy and radiotherapy [32-34]. We have been studying the molecular mechanism of the growth inhibitory effects of vesnarinone against the head and neck cancer cells, and we demonstrated that vesnarinone induces the expression of various kinds of genes such as p21^waf1 ^[35] and transforming growth factor β stimulated clone (tsc)-22 [22]. A previous phase study revealed that two partial responses against metastatic lesions in a patient with metastatic breast cancer involving lymph nodes, liver, and bone, and in a patient with metastatic non-small-cell lung cancer involving the brain and lymph node, when the patients were treated with vesnarinone and gemcitabine [34]. These clinical observations suggested the anti-metastatic activity of vesnarinone; however, the molecular mechanism of the anti-metastatic action of vesnarinone remained unclarified in those studies. Vesnarinone was shown to exhibit anti-proliferating effects at a dose of 200 mg/kg against a human adenoid squamous carcinoma-forming cell line grown in nude mice [23]. In order to specifically investigate the anti-metastatic effects of vesnarinone, we administrated a lower dose of vesnarinone (50 mg/kg) to tumor-bearing nude mice. As expected, vesnarinone did not inhibit tumor size in the masseter muscle (data not shown), but it did inhibit the lymph node metastasis. These results indicated that although vesnarinone specifically inhibits lymph node metastasis, this does not occur by inhibition of lymph node cell growth.

In the present study, we demonstrated the novel transcriptional regulation of CXCR4 via the transcription factor KLF2 in vesnarinone-treated oral cancer cells. It has been shown that the expression of CXCR4 is transcriptionally upregulated by several transcription factors such as c-Myc [36], NF-κB [37] and hypoxia inducible factor (HIF) [38], and is downregulated by Yin-Yang 1 [36]. In addition, cytokine signaling induced by granulocyte colony-stimulating factor [39], interferon gamma [40], and tumor necrosis factor-alpha [41] have also been reported to regulate CXCR4 expression. Among these transcription factors and cytokine signaling, vesnarinone-regulatory element located in the region between -300 and -167 from the transcriptional starting site contained one canonical NF-κB binding site. On the other hand, Manna and co-workers reported that vesnarinone suppresses the TNF-induced activation of NF-κB [42]. Thus, we initially expected to find that NF-κB is a vesnarinone-inducible transcriptional regulator for CXCR4. However, our site-directed mutagenesis study showed that vesnarinone did alter promoter activity in samples with either a mutated NF-κB site or that of samples with a wild-type NF-κB site (data not shown]. Moreover, our microarray analysis revealed that treatment with vesnarinone did not alter NF-κB subunit p50 and p65 mRNA levels. Thus, we concluded that NF-κB is not a vesnarinone-inducible CXCR4 regulator.

KLF2 [i.e., lung Krüppel-like factor (LKLF)], was originally identified through the use of the zinc finger domain of erythroid Krüppel-like factor (ELKF) as a hybridization probe; and KLF2 is expressed in a limited number of tissues, with the predominant expression seen in the lungs and spleen [43]. KLF2 is required to program the quiescent state of T cells via the downregulation of c-Myc [44]. Moreover, it has been demonstrated that KLF2 regulates diverse biological activities, such as adipogenesis [45], inflammation [46], cell growth [47], apoptosis [48], tumorigenesis [49], and angiogenesis [50]. In these processes, KLF2 upregulates p21^waf1 ^[47] and cytosolic phospholipase A2 alpha [49], and downregulates several types of molecules, including peroxisome proliferator-activated receptor-gamma [44], vascular cell adhesion molecule-1 [46], E-selectin [45], WEE1 [48], and vascular endothelial growth factor receptor 2 [50]. KLF2 is grouped with KLF1 and KLF4, which are characterized by amino-terminal acidic activation domains, inhibitory regions adjacent to the zinc fingers, and conserved nuclear localization signals, and which function both as activators as well as repressors [51]. However, abundant evidence suggested that KLF2 dominantly functions as a repressor. In the present study, KLF2 was also found to negatively regulate CXCR4 expression, most likely via the binding of a canonical CACCC motif. Since other KLF families are able to recognize this motif, CXCR4 may be downregulated by other KLF families. However, of the 13 members of the KLF family examined in the present microarray analysis (data not shown), only KLF2 mRNA was upregulated by 48-h treatment with vesnarinone. Thus, as regards the regulation of CXCR4 expression by vesnarinone, it is expected that KLF2 is the most important regulator among the member of KLF family that putatively binds to this CACCC motif.

In the present study, the mechanism of transcriptional upregulation of KLF2 by vesnarinone was still unclear. We have previously demonstrated that vesnarinone directly activates p21^waf1 ^gene promoter via Sp1 sites in a human salivary gland cancer cell line [34]. Since the 5'-flanking region of the KLF2 gene manifests typical features of GC-rich promoter with multiple transcription factor Sp1 binding sites [51], KLF2 might be also upregulated by vesnarinone via the activation of Sp1.

CXCR4 functions as a coreceptor for T cell line-tropic HIV-1 entry. Previously, vesnarinone was found to inhibit the replication of HIV-1 in a peripheral blood lymphocyte model via the inhibition of cytokine production [52]. Although vesnarinone was not developed as a targeted agent against CXCR4, it may have other targets against the metastasis of oral cancer. However, this effect might have been the result of the impaired expression of CXCR4 in peripheral blood lymphocytes due to treatment with vesnarinone. The findings of the present study suggest that vesnarinone might be useful as an anti-metastatic agent for patients with CXCR4-related oral cancer. Moreover, our clinical observations suggest that whereas vesnarinone does not exert dramatic anti-tumor effects, it does induce tumor differentiation and dormancy, effects previously shown to prolong the lives of patients with head and neck cancers [32,33]. Due to agranulocytosis and mild anti-tumor effects, care must be taken to administer vesnarinone as the sole agent as a first-line therapy to patients with OSCC. However, a combination of vesnarinone with conventional chemotherapy or radiotherapy might be used as a novel second- or third-line anti-metastatic intervention in patients with CXCR4-related OSCC.

## Conclusion

Our results indicated that vesnarinone downregulates CXCR4 expression via the upregulation of KLF2 in oral cancer.

## Abbreviations

OSCC: oral squamous cell carcinoma; SDF-1: stromal cell-derived factor-1; vesnarinone; 3,4-dihydro-6-[4-(3,4-dimethoxybenzoyl)-1-piperazinyl]-2-(1H)-quinolinone; CMC: carboxymethylcellulose; 5-FU: 5-fluorouracil; CDDP: cis-diamminedichloroplatinum; DMEM: Dulbecco's modified Eagle medium; GAPDH: glyceraldehyde 3-phosphate dehydrogenase.

## Competing interests

The authors declare that they have no competing interests.

## Authors' contributions

DU designed experiments, drafted manuscript and performed siRNA knock-down. TO performed luciferase assay and nude mice experiments. NMB performed bacterial culture, recombination of plasmid and purification of the plasmid. YT performed real-time PCR and flow cytometry. NK was responsible for cell cultures and participated in PCR analysis and data collection. TT and HN participated in discussion and manuscript preparation. YM conceived the study and revised the manuscript. All authors read and approved the final version of the manuscript.

## Supplementary Material

Additional file 1 **Downregulation of CXCR4 protein by vesnarinone**. ACC-M or Hela cells treated with or without chemotherapeutic agents were incubated with mouse IgG isotype control or with anti-CXCR4 monoclonal antibody. Then the cells were incubated with PE-labeled goat anti-mouse IgG and analyzed by flow cytometry in order to determine the expression of CXCR4 protein. The white zones show the cells treated with vesnarinone stained by mouse IgG isotype control. The black zones show cells treated with vesnarinone, and gray zones show the cells treated with 5-FU (*upper*) or CDDP (*lower*), respectively. The black zones and gray zones were stained by anti-CXCR4 monoclonal antibody. Click here for file

Additional file 2 **Inhibition of luciferase activity in the presence of vesnarinone**. Several CXCR4-luciferase constructs were transfected into B88 cells, which were treated with or without vesnarinone, and then luciferase activities were measured at the indicated time points. (a) -2632/+86, (b) -832/+86, (c) -300/+86, (d) -167/+86, (e) -72/+86, (f) -29/+86. Click here for file

Additional file 3 **Forced expression of KLF2 impaired CXCR4 promoter activity in ACC-M cells**. Stable transfectants of CXCR4 promoter-luciferase construct (-300/+86) were transfected with pcDNA3 or KLF2 expression vectors, and then luciferase activity was measured at the indicated time points. *, Significant as determined by one-way ANOVA (vs. pcDNA3, *P *< 0.05). Click here for file
