# Supplementary figures and images for: Vesnarinone downregulates CXCR4 expression via upregulation of Krüppel-like factor 2 in oral cancer cells
Source: Mol Cancer. 2009 Aug 12;8:62. doi: 10.1186/1476-4598-8-62 (PMC2738650; doi:10.1186/1476-4598-8-62)

**ACC-M**

**VES**   **5-FU**

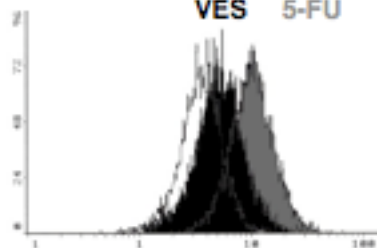

**VES**   **CDDP**

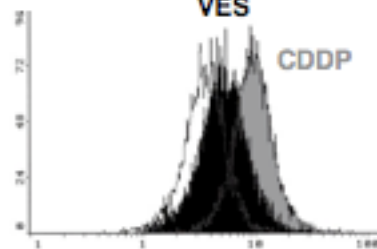

**CXCR4**

**Hela**

**VES**   **5-FU**

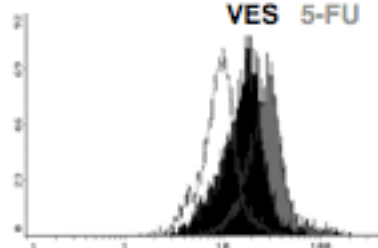

**VES**   **CDDP**

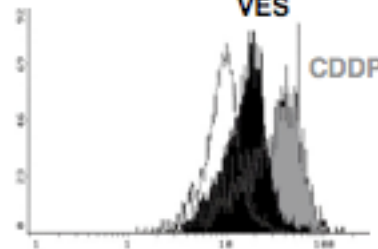

**CXCR4**

Supplement: Additional file 1 — Downregulation of CXCR4 protein by vesnarinone. ACC-M or Hela cells treated with or without chemotherapeutic agents were incubated with mouse IgG isotype control or with anti-CXCR4 monoclonal antibody. Then the cells were incubated with PE-labeled goat anti-mouse IgG and analyzed by flow cytometry in order to determine the expression of CXCR4 protein. The white zones show the cells treated with vesnarinone stained by mouse IgG isotype control. The black zones show cells treated with vesnarinone, and gray zones show the cells treated with 5-FU (upper) or CDDP (lower), respectively. The black zones and gray zones were stained by anti-CXCR4 monoclonal antibody. [file 1476-4598-8-62-S1.pdf]

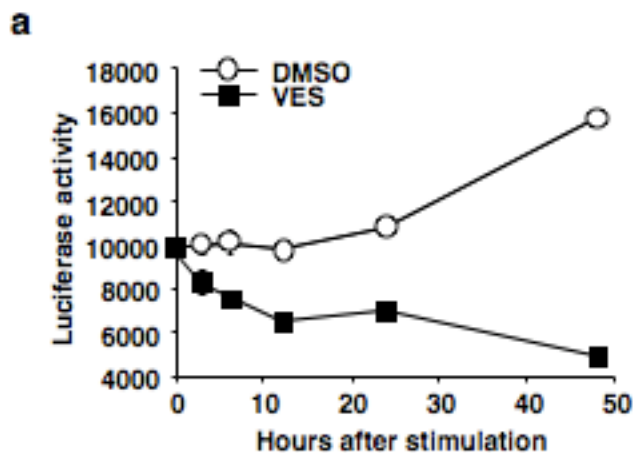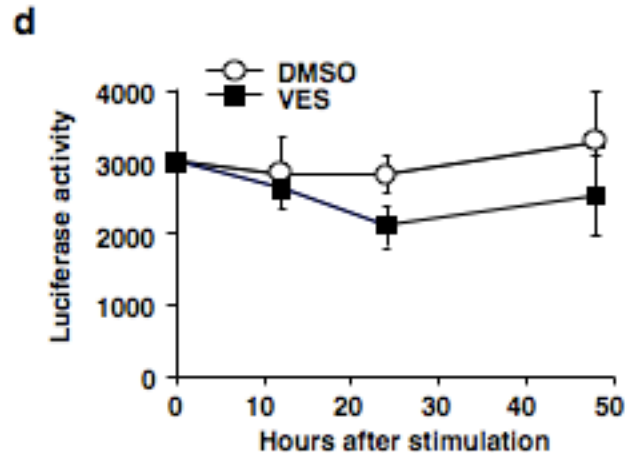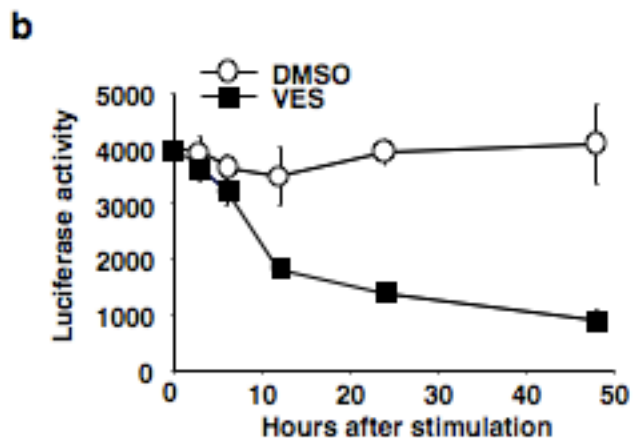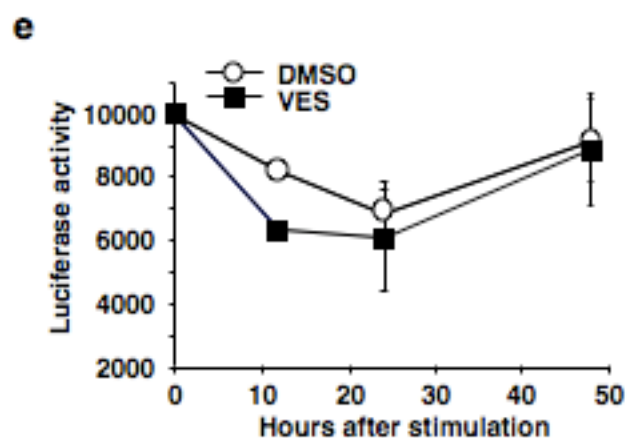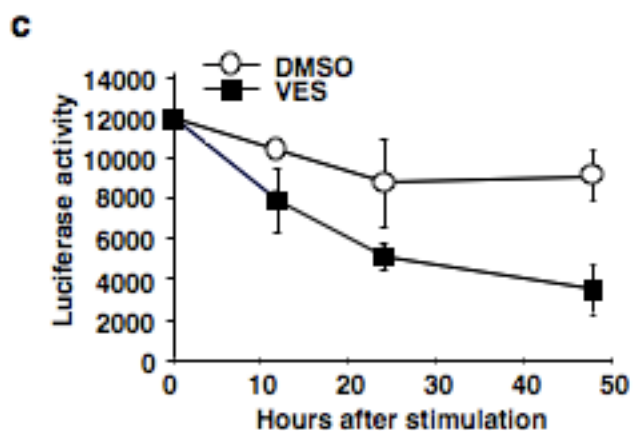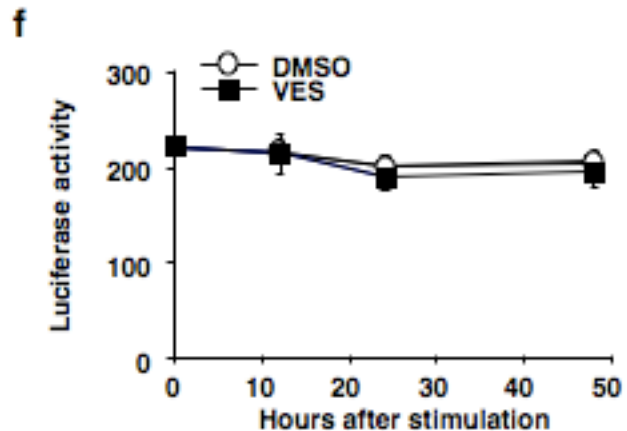

Supplement: Additional file 2 — Inhibition of luciferase activity in the presence of vesnarinone. Several CXCR4-luciferase constructs were transfected into B88 cells, which were treated with or without vesnarinone, and then luciferase activities were measured at the indicated time points. (a) -2632/+86, (b) -832/+86, (c) -300/+86, (d) -167/+86, (e) -72/+86, (f) -29/+86. [file 1476-4598-8-62-S2.pdf]

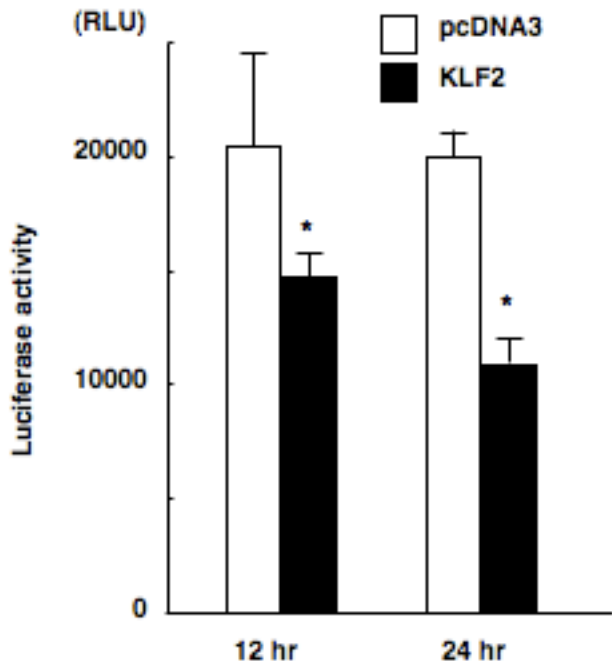

Supplement: Additional file 3 — Forced expression of KLF2 impaired CXCR4 promoter activity in ACC-M cells. Stable transfectants of CXCR4 promoter-luciferase construct (-300/+86) were transfected with pcDNA3 or KLF2 expression vectors, and then luciferase activity was measured at the indicated time points. *, Significant as determined by one-way ANOVA (vs. pcDNA3, P < 0.05). [file 1476-4598-8-62-S3.pdf]
